# Sustained weight loss in patients treated with mifepristone for Cushing’s syndrome: a follow-up analysis of the SEISMIC study and long-term extension

**DOI:** 10.1186/s12902-015-0059-5

**Published:** 2015-10-27

**Authors:** Henry G. Fein, T. Brooks Vaughan, Harvey Kushner, David Cram, Dat Nguyen

**Affiliations:** Johns Hopkins University School of Medicine and Sinai Hospital of Baltimore, 2435 W. Belvedere Avenue, Baltimore, MD 21215 USA; Division of Endocrinology, Diabetes & Metabolism, University of Alabama at Birmingham School of Medicine, BDB 706, 1720 2nd Avenue South, Birmingham, AL 35294 USA; BioMedical Computer Research Institute, 9743 Redd Rambler Place, Philadelphia, PA 19115 USA; Corcept Therapeutics, 149 Commonwealth Drive, Menlo Park, CA 94025 USA

**Keywords:** Cushing’s syndrome, Korlym, Long-term, Mifepristone, Obesity, SEISMIC, Weight loss

## Abstract

**Background:**

Overweight and obesity are common among patients with Cushing’s syndrome (CS) and may persist in some patients even after ostensibly curative surgery, contributing to cardiometabolic dysfunction and increased cardiovascular risk. Mifepristone, a selective glucocorticoid receptor antagonist, was effective in controlling hyperglycemia in a 24-week trial of adults (*N* = 50) with endogenous CS and associated type 2 diabetes mellitus/impaired glucose tolerance or hypertension who had failed or were not candidates for surgery (SEISMIC, Study of the Efficacy and Safety of Mifepristone in the Treatment of Endogenous Cushing’s Syndrome). This analysis examines long-term weight change among patients who received mifepristone in SEISMIC and enrolled in a long-term safety extension (LTE) study.

**Methods:**

Patients completing the 24-week SEISMIC study and subsequent 6-week off-drug safety evaluation were invited to enroll in the LTE study. Mifepristone doses at the end of SEISMIC were the LTE starting doses. Body weight measures were reviewed at baseline and week 24 of SEISMIC and at LTE month 6, 12, 18, 24, and final visit (last observation collected during the LTE study).

**Results:**

Of the 30 patients enrolled in the LTE, evaluable weight data were available for 29 (20/29 female; mean age of 44.7 ± 11.2 years). These patients received mifepristone for a median of 29.2 months (range 8.4–41.9). Mean ± SD weight from SEISMIC baseline to LTE final visit decreased by 10.3 ± 16.3 kg (mean 105.4 ± 34.3 kg to 95.1 ± 32.9 kg), a 9.3 % decrease from baseline weight (*P* = 0.0008). Of the 29 LTE patients, 18 (62.1 %) lost ≥5 % of body weight by the end of the initial 24-week treatment period; this ≥5 % weight loss persisted in 83.3 % (15/18) at LTE final visit. Ten patients (34.5 %) lost ≥10 % of initial body weight by week 24 of SEISMIC, which persisted in 80 % at LTE final visit. No new safety signals were detected with long-term mifepristone use.

**Conclusion:**

Clinically meaningful weight loss achieved during a 24-week study of mifepristone for CS persisted for two additional years in patients who remained on therapy. Long-term treatment with mifepristone appears to have a beneficial effect on weight in patients with endogenous CS.

**Trial Registration:**

NCT00569582 (SEISMIC); NCT00936741 (Long-Term Extension).

## Background

Endogenous Cushing’s syndrome (CS), a complex metabolic disorder resulting from prolonged exposure to elevated cortisol, is associated with substantial morbidity and mortality, particularly in relation to cardiovascular events [1–4]. Obesity is a prominent feature of CS, with 70 to 80 % of patients characterized as either overweight or obese [5–7]. Abdominal obesity contributes to insulin resistance, metabolic abnormalities, and cardiovascular risk in CS patients [6, 8, 9]. Although the mortality risk may decrease once remission of hypercortisolism is achieved, overall mortality and cardiovascular risk remain elevated in these patients when compared with control populations [4, 10]. Surgical resection of an adrenocorticotropic hormone (ACTH)- or cortisol-secreting tumor, which is the first-line treatment, often results in decreased body weight during early remission [11–13]. However, recent studies have shown that body weight can increase after initial improvement even if postoperative biochemical cure occurs [7, 8, 14, 15], which may be a contributing factor in the increased cardiovascular risk seen even where surgery was successful.

In patients with recurrent or persistent hypercortisolism, however—ie, in those for whom remission fails—mortality is increased compared with control populations (standardized mortality ratios 1.7–4.8) [3, 16, 17]. Given that long-term recurrence of hypercortisolism after initial surgical success following transsphenoidal pituitary surgery in patients with Cushing’s disease (CD) has been reported in up to 66 % of patients followed postoperatively for a mean duration of 14 years (range 1–37 years) [11, 18–22], recurrent disease is a major concern. Secondary treatment options for patients with recurrent or persistent endogenous CS, which may include pharmacologic therapy, are thus often utilized. Data on the long-term metabolic profiles of patients following pharmacologic treatments are needed.

Mifepristone (Korlym®, Corcept Therapeutics, Menlo Park, CA), a glucocorticoid receptor antagonist, was associated with significant improvements in hyperglycemia in the 24-week SEISMIC (Study of the Efficacy and Safety of Mifepristone in the Treatment of Endogenous Cushing’s Syndrome) trial; 15/25 (60 %) had a ≥25 % reduction in area under the curve for glucose and 21/40 (52.5 %) had either a ≥5 mmHg reduction in diastolic blood pressure or reduction in antihypertensive medications [23]. A significant reduction in mean body weight in patients with CS was also noted during the study. Patients who completed the SEISMIC study could elect to enroll in a long-term safety extension (LTE) study. This report presents follow-up data examining the persistent effects of mifepristone on weight parameters in patients from the SEISMIC study who subsequently entered the LTE study.

## Methods

Details of the study design and patient population of SEISMIC have been previously published [23]. SEISMIC was a 24-week, open-label, multicenter study of mifepristone administered as a once-daily oral dose to adults with confirmed endogenous CS who had type 2 diabetes mellitus, impaired glucose tolerance, or a diagnosis of hypertension [23] and had failed or were not candidates for surgery. Mifepristone was started at a dose of 300 mg/day, with titration to a maximum of 1200 mg/day based on investigator clinical discretion. At the end of SEISMIC, patients underwent a 6-week off-drug safety evaluation period. Of the 34 patients who completed SEISMIC, 30 elected to enroll in the LTE study. The starting dose in the LTE was the same as the final dose in SEISMIC, with further dose titration at the discretion of the treating clinical investigators. The study designs were approved by the Western Institutional Review Board (Puyallup, WA) and an institutional review board at each study center [23]. Written informed consent was obtained from all patients for both studies. Patients were free to discontinue from the studies at any time.

This analysis reviews weight assessments taken at baseline and week 24 of SEISMIC as well as at months 6, 12, 18, 24, and final visit (each patient’s last observation) of the LTE study. Waist circumference and body composition were assessed in SEISMIC, but not the LTE. Safety was assessed throughout the SEISMIC and extension studies via adverse events (AEs), vital signs, physical exams, and clinical laboratory tests.

### Statistical analysis

All numerically continuous data are presented as mean ± SD unless otherwise specified. The percentage change in body weight from baseline was evaluated across time points using a mixed-model repeated-measures analysis of variance. No imputations for missing data were performed. Weight loss was evaluated descriptively using a categorical loss of at least 5 % of the patient’s body weight. A Kaplan-Meier plot was constructed based on the time to achieve weight loss of ≥5 % in SEISMIC and throughout the LTE. A separate plot was constructed to show time to achieve weight loss of ≥10 %. Time was right censored for patients who did not complete the LTE. Weight loss persistence was defined as a loss of at least 5 % of body weight at week 24 of SEISMIC that was maintained to the indicated study visits in the LTE trial. An additional analysis evaluated weight loss of ≥10 % at week 24 of SEISMIC that was maintained at this threshold to the indicated study visits in the LTE trial. All P-values ≤0.05 were considered statistically significant and no Bonferroni corrections for simultaneous multiple inferences were performed. All analyses were performed using SAS version 9.2 software (SAS Institute, Cary, NC).

## Results

### Patients

**Fig. 1:**
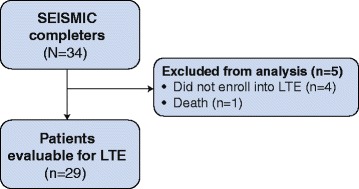
Participant flow. LTE: long-term extension

Of the 30 CS patients enrolled in the LTE (Fig. 1), evaluable body weight data were available for 29 patients (20 women and 9 men; mean age, 44.7 ± 11.2 years) (Table 1), who were treated with mifepristone at an average daily dose of 758 ± 290 mg. Twenty-six patients had CD and three patients had ectopic ACTH. Baseline biochemical status is reported in Table 2. Patients were treated in the LTE for a median of 29.2 months (range 8.4–41.9 months), and 25 patients received therapy for at least 2 years. At baseline in the SEISMIC study, six patients (20.7 %) were overweight (BMI 25–29) and 21 (72.4 %) were obese (BMI ≥30); nine had a BMI ≥40. The mean waist circumference was 122.4 ± 23.9 cm.

**Table 1:** Demographic and baseline characteristics of patients with evaluable body weight enrolled in the LTE study

Characteristic	LTE population (*n* = 29)
Sex, *n* (%)	
Male	9 (31)
Female	20 (69)
Race, *n* (%)	
White	24 (83)
Black or African American	5 (17)
Age, y	
Mean (SD)	44.7 (11.2)
Min, max	26, 71
Weight, kg	
Mean (SD)	105.4 (34.3)
Min, max	61.3, 198.7
BMI, kg/m^2^	
Mean (SD)	37.7 (11.7)
Min, max	24.1, 66.4
BMI category, *n* (%)	
< 25	2 (6.9)
25-29	6 (20.7)
≥ 30	21 (72.4)
≥ 40	9 (31)

*BMI* body mass index; *LTE* long-term extension

**Table 2:** Baseline biochemistry for patients with evaluable weight data enrolled in the LTE study

Variable, mean (SD)	Cushing’s disease (*n* = 26)	Ectopic ACTH (*n* = 3)	Overall (*n* = 29)
ACTH, pg/mL	54.5 (33.6)	180.0 (158.0)	67.5 (65.6)
24 h UFC, μg/24 h	144.9 (153.2)	3158.4 (3625.3)	456.6 (1353.5)
Serum cortisol, μg/dL	21.1 (5.8)	44.5 (16.9)	23.5 (10.2)
Late-night salivary cortisol, μg/dL	0.31 (0.32)	2.5 (2.4)	0.56 (1.0)

*ACTH* adrenocorticotropic hormone; *UFC* urinary free cortisol; *LTE* long-term extension

### Weight loss and change in body composition during SEISMIC and LTE

**Fig. 2:**
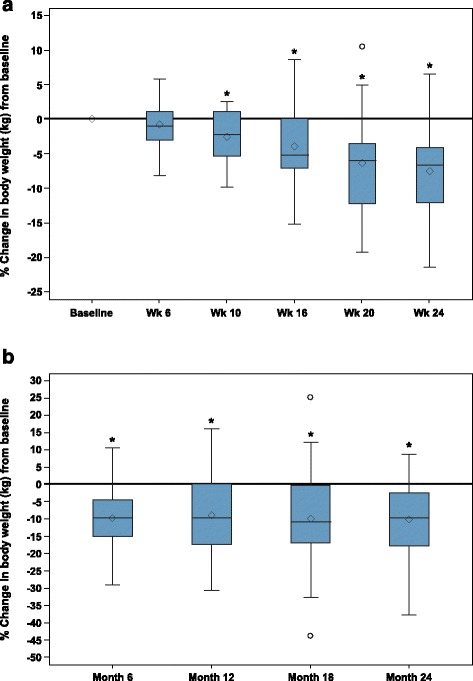
Mean percent change in body weight from baseline in SEISMIC by visit in **a** SEISMIC and by month in **b** LTE. *The changes from baseline were statistically significant from weeks 10–24 in the 24-week treatment period and at each time point in the long-term extension (*P* < 0.0001). Diamonds = means; horizontal lines within boxes = medians; ends of boxes = 25th/75th percentiles; “whiskers” = range of the min to the max but not longer than 1.5 times the interquartile range (IQR); circles = values beyond the 1.5 IQR. LTE: long-term extension

Among the 29 patients included in this analysis, mean waist circumference decreased by 9.3 cm in women and 8.3 cm in men from baseline to week 24 of SEISMIC. Mean percent total body fat declined by 3.7 % in women and 0.3 % in men, and percent trunk fat declined by 2.5 % in women and 1.2 % in men by week 24 of SEISMIC. On the other hand, mean total lean body mass increased by 3.9 % in women and 1.3 % in men by week 24 of SEISMIC. Percentage decreases in body weight from baseline were significant at weeks 10–24 of SEISMIC (all *P* < 0.0001) and at each assessment in the LTE (all *P* < 0.0001) (Fig. 2). A 7.5 % decrease in weight was observed from baseline to week 24 (*P* < 0.0001). At the 24-week visit during SEISMIC, 62.1 % (18/29) of patients had lost ≥5 % of baseline body weight. An additional analysis found 34.5 % (10/29) of patients lost ≥10 % of baseline body weight at the end of SEISMIC.

**Fig. 3:**
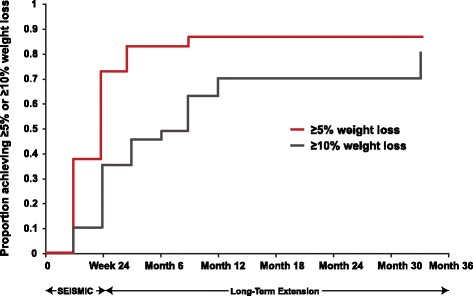
Kaplan-Meier plot for proportion of patients achieving ≥5 and ≥10 % weight loss in SEISMIC and the LTE. LTE: long-term extension

By the end of the LTE, 86 % (25/29) achieved ≥5 % weight loss, while 72 % (21/29) achieved ≥10 % weight loss. The crude rate for the 10 % weight loss was lower than the life-table estimate observed in the Kaplan-Meier plot (Fig. 3, 80 % for ≥10 % weight loss). A 9.3 % decrease in weight was observed from baseline to final visit in the LTE (*P* = 0.0008) (Table 3).

**Table 3:** Persistence of weight loss in SEISMIC and the LTE

	SEISMIC		LTE		
Variable	Baseline (*n* = 29)	Week 24 (*n* = 29)	Month 6 (*n* = 27)	Month 18 (*n* = 25)	Final visit (*n* = 29)
Weight, kg					
Mean ± SD	105.4 ± 34.3	97.2 ± 30.8	95.2 ± 32.2	96.6 ± 34.7	95.1 ± 32.9
Change weight	**—**	−8.2 ± 9.3	−10.5 ± 12.3	−11.4 ± 18.7	−10.3 ± 16.3
% Change in weight	**—**	−7.5 ± 7.1	−9.7 ± 9.7	−10.0 ± 14.4	−9.3 ± 13.4
BMI, kg/m^2^					
Mean ± SD	37.7 ± 11.7	34.7 ± 10.0	34.3 ± 9.7	34.0 ± 9.7	33.7 ± 9.9
BMI category, *n* (%)					
≥ 30	21 (72.4)	17 (58.6)	16 (59.3)	16 (64.0)	18 (62.1)
≥ 40	9 (31.0)	7 (24.1)	6 (22.2)	4 (16.0)	6 (20.7)

Final visit is defined as the last post-entry observation collected during the LTE study

*BMI* body mass index; *LTE* long-term extension

### Persistence of weight loss from SEISMIC across LTE

Of the 18 patients who lost ≥5 % of body weight by the end of the 24-week treatment period, 83.3 % (*n* = 15) maintained ≥5 % weight loss at final visit in the LTE study (Table 4). Twelve of the 18 patients lost additional weight in the LTE (7 lost ≥5 %, 5 lost <5 %). The six patients with weight gain during the LTE still had an average net loss of 3.23 % (1.9 ± 4.4 kg) when compared to baseline in SEISMIC. Of the ten patients who lost ≥10 % of body weight during SEISMIC, 80 % maintained that degree of weight loss by the LTE final visit.

**Table 4:** Categorical weight loss in SEISMIC and persistence during the LTE study

Categorical weight loss in SEISMIC	Time point in LTE study	Weight loss persistence^a^, *n* (%)
≥5 % weight loss (*n* = 18)^b^	Month 6	14 (82.4)
	Final visit^c^	15 (83.3)
≥10 % weight loss (*n* = 10)^b^	Month 6	8 (80)
	Final visit^c^	8 (80)

^a^Persistence is defined as maintenance of the weight loss threshold in SEISMIC to LTE assessment time points. ^b^One subject was excluded from this analysis because the only post-baseline weight was assessed on the day of death, which occurred in the hospital in relation to a serious adverse event deemed not related to study drug by the investigator. ^c^Final visit is defined as the last post-entry observation collected during the LTE study

*LTE* long-term extension

### Safety

All patients (*n* = 29) reported at least one AE during the LTE; the most common AEs reported were nausea (52 %), decreased blood potassium (48 %), fatigue (45 %), headache (38 %), and endometrial thickening (35 %). Three patients discontinued from the study because of AEs (*n* = 1 each: adrenal insufficiency, endometrial thickening, endometrial disorder). During the LTE, the term “adrenal insufficiency” was used to describe the events experienced by five patients. Three of these events were associated with co-existing infections. The symptoms of “adrenal insufficiency” were effectively managed with interruption of mifepristone and administration of dexamethasone in four patients and interruption of mifepristone without glucocorticoid supplementation in one patient. Severe hypokalemia (serum potassium ≤2.5 mEq/L) was reported in four patients, which resolved with treatment that included potassium supplements and mineralocorticoid antagonists. No patients discontinued from the LTE because of hypokalemia.

## Discussion

In CS, hypercortisolism can promote cardiometabolic abnormalities similar to that of metabolic syndrome, including increased abdominal fat, hypertension, diabetes mellitus, and hyperlipidemia [6, 8, 24, 25], which contribute to the increased cardiovascular risk and mortality in these patients [2–4]. Terzolo et al. recently examined cardiovascular risk among patients with CS, followed at least 12 months postoperatively [10]. Patients with persistent disease following surgery (*n* = 24) continued to have elevated rates of hypertension (79 %), diabetes (54 %), central obesity (77 %), and elevated triglycerides (54 %) after 12 months, with little change compared to rates at diagnosis. Among patients in remission (*n* = 51), the rate of hypertension decreased by 41 %, central obesity decreased by 37 %, diabetes decreased by 17 %, and elevated triglycerides decreased by 16 % compared with diagnosis. However, despite the improvements following resolution of hypercortisolism, the rates of central obesity and elevated triglycerides remained significantly higher than the control population (45 vs 13 %; *P* = 0.0002 and 25 vs 5 %; *P* = 0.005, respectively). Therefore, increased emphasis is needed to address CS-related comorbidities, including cardiovascular risk, before and after remission of hypercortisolism is achieved, as noted in recent CS guidelines [17]. However, long-term data will be needed to determine if improvement in cardiovascular risk factors in patients with CS will lead to a further reduction in mortality.

While cardiovascular risk was not formally assessed in the 6-week SEISMIC trial, treatment with mifepristone was shown to improve glucose parameters in patients with CS that were refractory to other therapies and who had associated type 2 diabetes mellitus, impaired glucose tolerance, or hypertension [23, 26]. Walia et al. further demonstrated that large improvements in glucose tolerance and insulin sensitivity occurred during the first 6 weeks of mifepristone treatment [26] and continued to improve as beneficial changes in weight and waist circumference were attained at week 24. Our current study demonstrated that weight loss of ≥5 % is maintained and can persist for up to 3.5 years of mifepristone treatment. A weight loss of 5–10 % has been shown to reduce cardiovascular risk factors in other at-risk populations [27, 28]. For example, a randomized, multicenter trial of obese and overweight patients with type 2 diabetes found that a weight loss of 5 to <10 % after 1 year of intensive lifestyle intervention was associated with a statistically significant improvement in cardiovascular risk factors including reductions in glycated hemoglobin (A_1c_) and blood pressure [27].

Long-term therapy with mifepristone in patients with CS was associated with an AE profile comparable to that reported in the 24-week SEISMIC study [23]. There were five reported cases of “adrenal insufficiency” during the LTE, and all cases resolved with temporary drug interruption with or without supplemental glucocorticoid administration. Yuen et al. [29] recently described how symptoms of “excessive glucocorticoid receptor antagonism” associated with mifepristone can resemble some symptoms of adrenal insufficiency (eg, nausea, fatigue, vomiting, and low appetite). However, serious symptoms of adrenal crisis, such as hypotension or hyperkalemia, are unlikely to occur due to the rise in cortisol levels and subsequent activation of the mineralocorticoid receptors during mifepristone therapy. If clinical signs or symptoms of excessive glucocorticoid receptor antagonism are suspected, therapy with mifepristone should be discontinued temporarily, and in some cases, administration of supplemental glucocorticoids may be useful. Treatment can be resumed at a lower dose once signs and symptoms of excessive glucocorticoid receptor antagonism are resolved.

This follow-up analysis of clinical trial data contributes useful information on the long-term use of mifepristone and weight loss in patients with CS. Other cardiometabolic parameters, such as glycemic control, waist circumference, and blood pressure, were not routinely assessed during the LTE, nor did this study control for patient-specific factors such as diet, physical activity, or other lifestyle modifications. Likewise, the influence of disease etiology on weight outcomes was not examined because of the limited number of patients that enrolled in the LTE with diagnoses other than CD. Additional studies in patients with CS to assess long term metabolic and cardiovascular benefits associated with sustained weight loss from cortisol blockade with mifepristone are warranted. It remains to be determined whether sustained improvement in cardiovascular risk factors will translate to reduced mortality in patients with CS.

## Conclusion

A follow-up analysis of clinical trial data found clinically meaningful weight loss (≥5 % of body weight) achieved during short-term mifepristone use was sustained in approximately 80 % of patients with CS who were treated with mifepristone for up to 3.5 years. No new safety signals were detected with long-term mifepristone use.

## Abbreviations

ACTH: Adrenocorticotropic hormone
BMI: Body mass index
CD: Cushing’s disease
CS: Cushing’s syndrome
LTE: Long-term extension
SEISMIC: Study of the Efficacy and Safety of Mifepristone in the Treatment of Endogenous Cushing’s Syndrome
